# Chemoreflex function and brain blood flow during upright posture in men and women

**DOI:** 10.14814/phy2.13571

**Published:** 2018-01-15

**Authors:** Christopher Hazlett, Heather Edgell

**Affiliations:** ^1^ School of Kinesiology and Health Sciences York University Toronto Ontario Canada; ^2^ Muscle Health Research Centre York University Toronto Ontario Canada

**Keywords:** Hemodynamics, menstrual cycle, orthostatic stress

## Abstract

Orthostatic intolerance is more common in women than men, and some studies have found that women in the early follicular (EF) phase of the menstrual cycle experience the greatest feelings of lightheadedness. Chemoreflex function while supine or upright was investigated to determine the potential contribution of ventilatory control to these phenomena. Men (*n* = 13) and women (*n* = 14) were tested while supine and 70° upright (head‐up tilt [HUT]) and given: (1) normoxia or (2) hypercapnia (5% CO_2_). Women were tested during the EF phase (days 2–5) and the midluteal phase (ML; days 18–24). During HUT, all groups reduced cerebrovascular resistance index (men: 1.45 ± 0.08 to 1.42 ± 0.07 mmHg/(cm·sec), EF: 1.38 ± 0.11 to 1.26 ± 0.10 mmHg/(cm·sec), ML: 1.25 ± 0.07 to 1.09 ± 0.07 mmHg/(cm·sec); *P* ≤ 0.019); however, only men increased ventilation (men: 11.99 ± 0.65 to 13.24 ± 0.83 L/min; *P* < 0.01). In response to hypercapnia in the supine position, men had a smaller increase of diastolic middle cerebral artery velocity compared to women in the ML phase (men: +9.1 ± 2.0 cm/sec, ML: +15.7 ± 3.1 cm/sec, *P* = 0.039). During hypercapnia in HUT (compared to hypercapnia while supine), all groups had an augmented increase of ventilation (men: +7.46 ± 1.34 vs. +5.84 ± 1.09 L/min, EF: +6.71 ± 0.83 vs. +5.48 ± 0.66 L/min, ML: +7.99 ± 1.13 vs. +5.65 ± 0.81 L/min; *P* ≤ 0.028), suggesting that all groups experienced augmentation of the CO_2_ chemoreflex; however, only men had an augmented increase of mean arterial pressure (+0.10 ± 0.58 to +4.71 ± 0.87 mmHg; *P* ≤ 0.017). Our results indicate that men have different ventilatory responses to upright tilt compared to women, and that the CO_2_ chemoreflex response is enhanced in upright posture in both sexes. Furthermore, sexually dimorphic blood pressure responses to this chemoreflex enhancement are evident.

## Introduction

Orthostatic intolerance is more prevalent in young women compared to men (Convertino 1998; Ganzeboom et al. 2003; Waters et al. 2005). Contributing factors likely include greater reductions in stroke volume (SV), cardiac output, venous return, and mean arterial pressure (MAP) during orthostatic stress in women (Convertino 1998; Jarvis et al. 2010; Edgell et al. 2012). Changes in ventilation and its control could also play a role due to potential influences on venous return and arterial carbon dioxide levels, thus influencing cardiac output and brain blood flow.

Peggs et al. (2012) observed that during orthostatic stress, women experience greater light headedness during the early follicular phase (EF; low estrogen and low progesterone) of their menstrual cycle compared to the midluteal phase (ML; high estrogen and high progesterone). Interestingly, women in the EF phase also have attenuated sympathetic nerve activity compared to women in the ML phase during orthostatic stress (Fu et al. 2009). Sympathetic output during orthostatic stress can be influenced by many autonomic reflexes including baroreceptors, mechanoreceptors, metaboreceptors, and chemoreceptors. Therefore, the contribution of each reflex to total sympathetic outflow during orthostatic stress should be investigated particularly in light of the fact that reflex function can change throughout the menstrual cycle. For example, during supine rest, ventilation is higher, despite lower arterial CO_2_, in the ML phase compared to women in the EF phase suggesting greater CO_2_ chemoreceptor activity during the ML phase (Slatkovska et al. 2006). If women in the EF phase retain lower CO_2_ chemoreceptor reactivity compared the ML phase during upright posture (or exhibit further attenuation), this could help to explain lower sympathetic activity in the EF phase during tilt.

Lower orthostatic tolerance in women could also be due to changes in brain blood flow responses. Brain blood flow is known to increase with higher arterial CO_2_ (Ito et al. 2003), and women have been shown to have increased cerebrovascular CO_2_ reactivity compared to men (Kastrup et al. 1997). However, Kastrup et al. (1997) concurrently used hyperoxia with hypercapnia (95% O_2_ with 5% CO_2_) which could have obscured the results. More recently, Peltonen et al. (2015) found no difference between the sexes when investigating the brain blood flow response to hypercapnia in men versus women in the EF phase of the menstrual cycle (low estrogen and low progesterone). Furthermore, a second study by Peltonen et al. (2016) investigated cerebrovascular conductance while comparing the EF phase to the late follicular phase (comparing low estrogen to high estrogen, respectively) and they found no difference between phases. To our knowledge, no studies have investigated the cerebrovascular response to hypercapnia between women in the ML phase (high estrogen and high progesterone) and either the EF phase (low estrogen and low progesterone) or men in order to investigate the role of combined estrogen and progesterone.

We hypothesized that: (1) during upright tilt, women would have lower MAP, lower brain blood velocity, and lower respiratory rate compared to men (therefore contributing to lower orthostatic tolerance); (2) women in the ML phase would have greater cerebrovascular reactivity to CO_2_ compared to EF; (3) CO_2_ chemoreflex function would be enhanced in the upright posture compared to supine in both sexes; and (4) women during the EF phase would have enhanced CO_2_ chemoreflex activity during tilt compared to the ML phase and men.

## Methods

### Ethical approval

Informed consent was obtained in writing and procedures were approved by the Office of Research Ethics at York University and conformed to the guidelines contained in the Declaration of Helsinki.

### Participant description

Participants did not have previously diagnosed cardiovascular or respiratory disease/dysfunction and women were not taking any oral contraceptives for at least 1 month prior to testing. All subjects were asked to refrain from smoking, heavy exercise, the consumption of fatty/processed foods, as well as caffeinated and alcoholic beverages 12 h prior to testing. Men (*n* = 13) were tested once, while women (*n* = 14) were tested twice, once during the EF phase (days 2–5) and once during the ML phase (days 18–24) of their menstrual cycle (Table 1). Cycle was determined by self‐report with guidance from the researchers.

**Table 1:** Participant anthropometrics

	Men	Women	
		EF	ML
*n*	13	14	
Age (years)	22.8 ± 1.1	22.8 ± 0.8	
Height (cm)	174.0 ± 1.7^a^	159.6 ± 1.3	
Body mass (kg)	74.9 ± 2.9^a^	60.9 ± 2.8	60.1 ± 2.7
Predicted *V*O_2max_ (mL/(kg·min))	53.0 ± 1.1^a^	41.1 ± 1.3	
BMI (kg/m^2^)	24.7 ± 0.7	24.0 ± 1.2	23.6 ± 1.0
FEV_1_ (%)	81.7 ± 2.0	84.0 ± 2.2	

EF, early follicular phase; ML, midluteal phase; *V*O_2max_, predicted maximal oxygen consumption; BMI, body mass index; FEV_1_, forced expiratory volume in 1‐sec test.

a Main sex effect (men vs. women).

### Measurements

Heart rate was continuously calculated from a standard ECG. Beat‐by‐beat continuous blood pressure and cardiac output were recorded using a noninvasive finger cuff (Finometer, Finapres Medical Systems). Blood pressure was calibrated between trials using a BPTru device. SV (mL/beat) was calculated as a quotient of *Q* and HR. Total peripheral resistance (TPR; mmHg/(L·min)) was calculated as a quotient of MAP (mmHg) and *Q*. Cardiac output (*Q*_i_) and therefore stroke volume (SV_i_) and total peripheral resistance (TPR_i_) were normalized to body surface area (Dubois and Dubois formula).

A transcranial Doppler system (Multigon Industries Inc.) was used to quantify middle cerebral artery (MCA) velocity using a 2‐MHz probe secured in position at the right temporal window. Cerebral perfusion pressure (CPP) was calculated as MAP × (distance from TCD probe to heart × 0.7355 mmHg/cmH_2_O). Cerebrovascular resistance index (CVR_i_) was calculated as CPP/mean MCA velocity. Resistance index was calculated as (systolic MCA velocity − diastolic MCA velocity)/systolic MCA velocity. Pulsatility index was calculated as (systolic MCA velocity − diastolic MCA velocity)/mean MCA velocity.

Spirometry was collected by a heated, linear pneumotachometer (Hans Rudolph, Series 3813). Tidal volume (*V*_t_; L) was calculated from volume inspired while breathing rate was calculated as the rate of inspiration. Ventilation (*V*_e_; L/min) was calculated as a product of breathing rate (breaths/min) and *V*_t_. End‐tidal oxygen (ETO_2_; mmHg) and carbon dioxide (ETCO_2_; mmHg) levels were measured via O_2_ and CO_2_ analyzers (Vacumed, Model 17620/17630). Self‐reported physical activity levels and frequency were recorded and used as a prediction of cardiorespiratory fitness using the Ainsworth equation to obtain an index of *V*O_2max_ (Ainsworth et al. 1992).

All signals obtained were relayed to a PowerLab data acquisition system (ADInstruments, PowerLab 16/35) and Labchart software (ADInstruments, Version 8.1.3).

### Protocol

**Figure 1:**
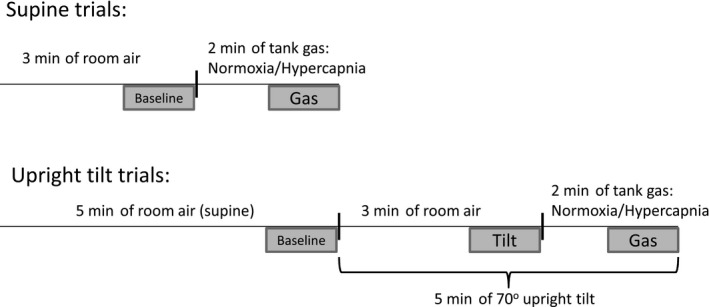
Timeline of supine and upright tilt trials. Shaded areas below the axes are time points when data were averaged for analysis.

Each test comprised a total of four randomized trials, two of which were in the supine position (normoxia and hypercapnia) and two in 70° head‐up tilt (HUT; normoxia and hypercapnia; Fig. 1). Trials were separated by at least 5 min. A manual blood pressure was taken between trials to ensure a return to baseline prior to starting a new trial. Supine trials consisted of 3 min of breathing room air/baseline followed by 2 min of gas administration. Tilted trials consisted of 5 min of breathing room air/baseline in the supine position followed by 70° HUT for 3 min while breathing room air. Then while still in the HUT position, gases were administered for 2 min (Fig. 1). Gases from compressed cylinders were humidified (Fisher & Paykel Healthcare, HC 150 Ambient Tracking) prior to breathing. Gases included: (1) hypercapnia (5% CO_2_, 21% O_2_, nitrogen balance) and (2) normoxia (0.03% CO_2_, 21% O_2_, nitrogen balance). Participants were blinded to which gas was being administered. Test termination criteria included a drop in systolic blood pressure to <70 mmHg or if the participant experienced symptoms of presyncope. There were no participants that reached this endpoint.

### Statistics and data analysis

**Figure 2:**
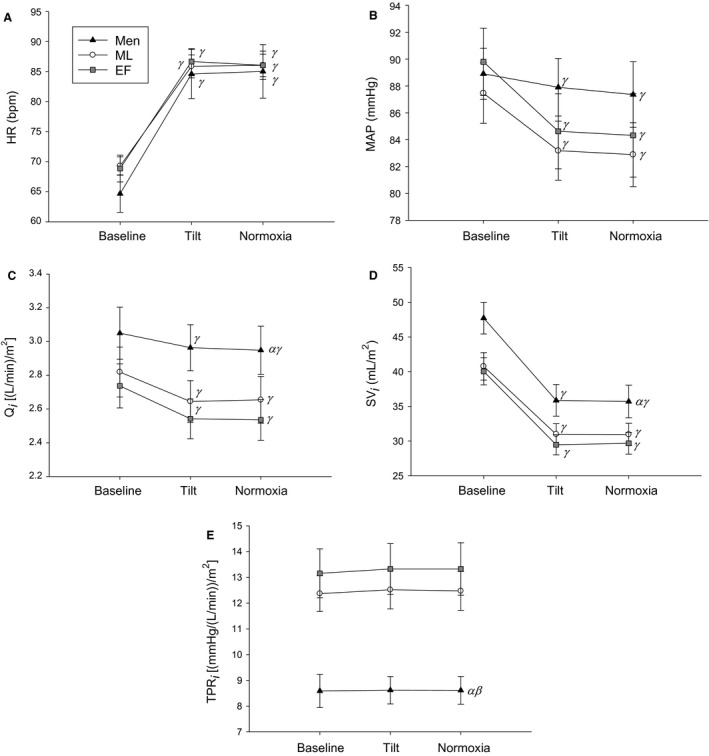
Heart rate (HR; A), mean arterial pressure (MAP; B), cardiac output index (*Q*_i_; C), stroke volume index (SV_*i*_; D), and total peripheral resistance index (TPR_i_; E) responses to tilt and normoxia in the tilted position. EF, early follicular phase; ML, midluteal phase. *α* indicates a main group effect (men vs. EF), *β* indicates a main group effect (men vs. ML), and *γ* indicates a significant difference from baseline. Men are black triangles, ML are white circles, and EF are gray squares.

**Figure 3:**
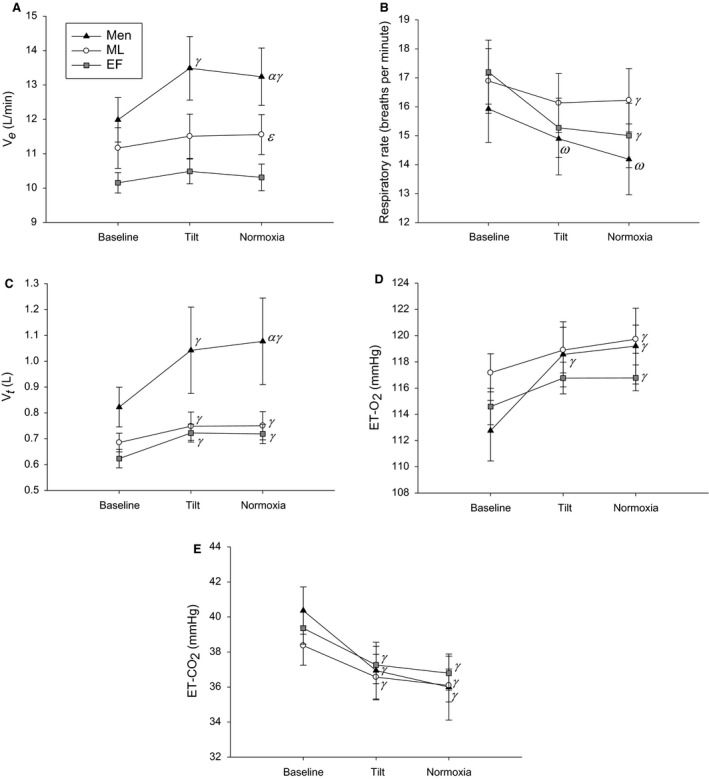
Ventilation (*V*_e_; A), Respiratory Rate (B), Tidal Volume (*V*_t_; C), End‐tidal Oxygen (ET‐O_2_; D), and End‐tidal Carbon Dioxide (ET‐CO_2_; E) responses to tilt and normoxia in the tilted position. EF is the early‐follicular phase, ML is the mid‐luteal phase. *α* indicates a main group effect (men vs. EF), *β* indicates a main group effect (men vs. ML), *ε* indicates a main phase effect (ML vs. EF), *ω* indicates a main effect of time when compared against EF, and *γ* indicates a significant difference from baseline. Men are black triangles, ML are white circles and EF are grey squares.

**Figure 4:**
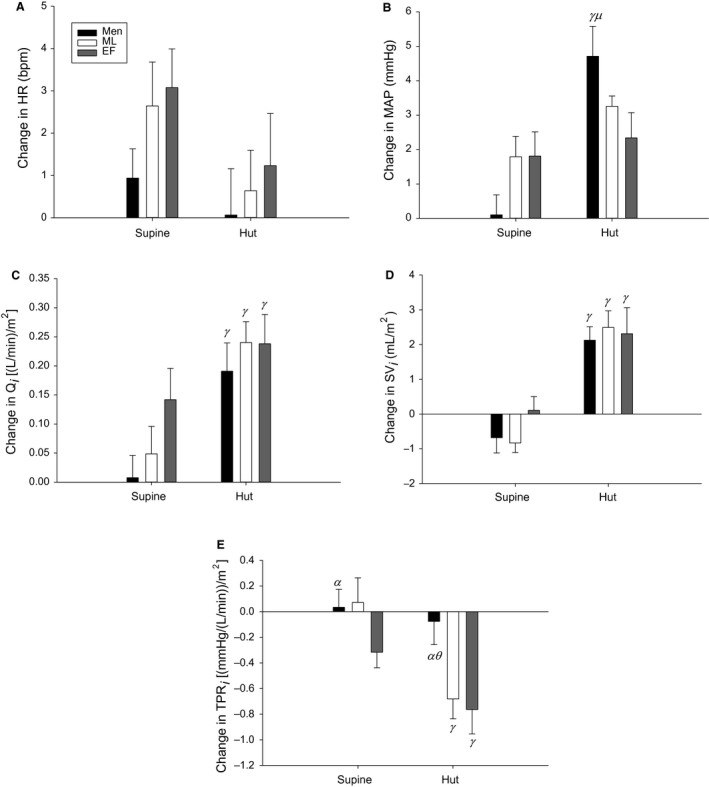
Changes in heart rate (HR; A), mean arterial pressure (MAP; B), cardiac output index (*Q*_*i*_; C), stroke volume index (SV_i_; D), and total peripheral resistance index (TPR_*i*_; E) in response to hypercapnia in the supine and 70° head‐up tilted (HUT) positions. EF is the early‐follicular phase, ML is the mid‐luteal phase. *α* indicates a main group effect (men vs. EF), *ϴ* indicates a main effect of time when compared against ML, *μ* indicates a group effect between men and EF during HUT, and *γ* indicates a significant difference from baseline. Men are black bars, ML are white bars and EF are grey bars.

**Figure 5:**
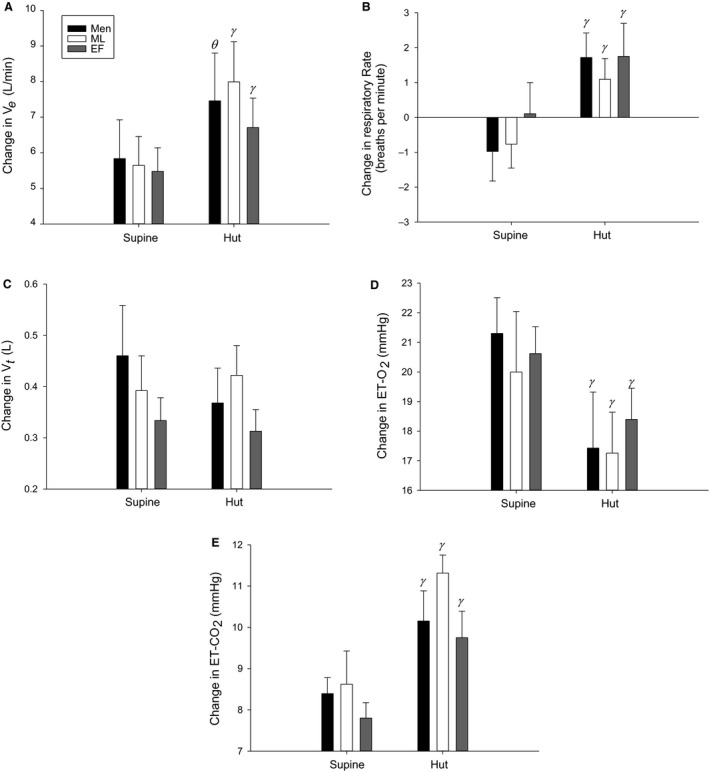
Changes in ventilation (*V*_e_; A), respiratory rate (B), tidal volume (*V*_t_; C), end‐tidal oxygen (ET‐O_2_; D), and end‐tidal carbon dioxide (ET‐CO_2_; E) in response to hypercapnia in the supine and 70° head‐up tilted (HUT) position. EF is the early‐follicular phase, ML is the mid‐luteal phase. *ϴ* indicates a main effect of time when compared against ML and *γ* indicates a significant difference from baseline. Men are black bars, ML are white bars and EF are grey bars.

All trials were randomized and results were analyzed using data analysis software (Sigmaplot, Version 12.0). One‐minute averages were analyzed at the last minute of “Baseline,” “Tilt,” and “Gas” time points as indicated by the gray‐shaded areas on Figure 1. For Figures 2 and 3, “Baseline,” “Tilt,” and “Gas” time points are used from the upright tilt trials. For Figures 4 and 5, in the supine trials, averages presented are the difference in “Gas” and “Baseline.” In the tilted trials, averages presented are the difference in “Gas” and “Tilt” (Fig. 1). Responses were compared between the supine and tilted trials within each gas (i.e., supine hypercapnia vs. tilted hypercapnia). Sex differences were investigated between men and each phase of the menstrual cycle by using separate two‐way repeated measures ANOVAs (sex and posture [repeated] as factors). Menstrual cycle effects were investigated using a two‐way repeated measures ANOVA (menstrual phase [repeated] and posture [repeated] as factors). Post hoc analysis of interaction effects used Tukey's HSD test to determine which groups statistically differed from one another. Significance was accepted at *P* < 0.05 and data are presented as mean ± SE.

## Results

Men were taller, heavier, and had a higher predicted *V*O_2max_ compared to women. Men and women were the same age and had the same body mass index and forced expiratory volume in 1 sec (Table 1).

### Supine normoxia

Compared to free breathing of room air, the normoxic gas administration in the supine position resulted in higher TPR_i_ in men (only when compared against the EF phase; 8.9 ± 0.7 to 9.0 ± 0.7 mmHg/(L·min·m^2^), *P* = 0.012 [main effect of gas administration], data not shown) and lower respiratory rate in men (16.7 ± 1.1 to 14.9 ± 1.1 breaths/min, *P* ≤ 0.040 [main effect of gas administration], data not shown). Women in both phases of the menstrual cycle had higher ET‐O_2_ (EF: 114.±1.5 to 116.8 ± 1.8 mmHg; ML: 118.9 ± 1.7 to 120.1 ± 1.4 mmHg; *P* = 0.009 [main effect of gas administration], data not shown) and lower ET‐CO_2_ (EF: 39.2 ± 1.1 to 38.5 ± 1.3 mmHg; ML: 37.1 ± 1.1 to 36.7 ± 1.0 mmHg; *P* = 0.05 [main effect of gas administration], data not shown) during normoxia administration compared to free breathing of room air. Men (when compared against the EF phase) had higher ET‐O_2_ during normoxia administration compared to free breathing of room air (114.7 ± 1.6 to 118.7 ± 3.3 mmHg, *P* = 0.028 [main effect of gas administration], data not shown).

### Normoxic tilt

Compared to baseline, all groups increased HR during tilt alone and during normoxia administration while tilted (Fig. 2A). Similarly, all groups had lower MAP during tilt alone and during normoxia administration while tilted compared to baseline (Fig. 2B). Men had higher *Q*_i_ compared to women during EF, yet all groups decreased *Q*_i_ during tilt alone and during normoxia administration while tilted compared to baseline (Fig. 2C). Men had higher SV_i_ compared to EF (Fig. 2D), yet SV_i_ decreased in all groups during tilt alone and during normoxia administration while tilted compared to baseline (Fig. 2D). Men had significantly lower TPR_i_ compared to women during both phases (Fig. 2E).

Only men increased their *V*_e_ during tilt alone and during normoxia administration while tilted (Fig. 3A). Men had higher *V*_e_ compared to EF, with women in the ML phase having higher *V*_e_ compared to the EF phase (Fig. 3A). Women during both phases had lower respiratory rates during normoxia administration while tilted compared to baseline (Fig. 3B). Men had lower respiratory rates during tilt alone and during normoxia administration while tilted compared to baseline but only when compared against EF (Fig. 3B). All groups increased *V*_t_ during tilt alone and during normoxia administration while tilted compared to baseline, and men had larger *V*_t_ compared to EF (Fig. 3C). Men increased ETO_2_ levels during tilt alone and during normoxia administration while tilted compared to baseline, and women in both phases had higher ETO_2_ during normoxia administration while tilted compared to baseline (Fig. 3D). All three groups had lower ETCO_2_ levels during tilt alone and during normoxia administration while tilted compared to baseline (Fig. 3E).

All groups had lower mean, systolic, and diastolic MCA velocity in response to tilt alone and during normoxia administration while tilted compared to baseline (Table 2). Women during both phases of their cycle decreased their CVR_i_ during tilt alone and during normoxia administration while tilted compared to baseline. Men decreased their CVR_i_ during tilt alone, but it was only lower during normoxia administration while tilted when compared against women during ML (Table 2). All groups had lower CPP during tilt alone and during normoxia administration while tilted compared to baseline (Table 2). Men had lower mean and diastolic MCA velocities and higher CVR_i_ compared to ML (Table 2).

**Table 2:** Cerebral hemodynamics during normoxic HUT

	Men			Women					
				EF			ML		
	Baseline	HUT	Normoxia	Baseline	HUT	Normoxia	Baseline	HUT	Normoxia
MCA_mean_ (cm/sec)	63.5 ± 3.5	54.8 ± 3.0^a^	53.5 ± 3.1^a^^,^ b	68.6 ± 3.9	61.6 ± 3.6^a^	60.0 ± 3.6^a^	71.4 ± 3.2	63.5 ± 2.6^a^	66.1 ± 4.3^a^
MCA_systolic_ (cm/sec)	97.3 ± 4.9	87.4 ± 4.4^a^	85.3 ± 4.2^a^	100.1 ± 4.9	90.7 ± 4.6^a^	89.7 ± 4.4^a^	105.1 ± 4.7	93.6 ± 3.7^a^	98.9 ± 6.0^a^
MCA_diastolic_ (cm/sec)	46.0 ± 2.7	39.8 ± 2.6^a^	39.1 ± 2.5^a^^,^ b	49.0 ± 3.0	45.4 ± 2.8^a^	43.7 ± 2.8^a^	50.2 ± 2.6	46.8 ± 2.3^a^	48.1 ± 3.3^a^
CVR_i_ (mmHg/(cm·sec))	1.45 ± 0.08	1.40 ± 0.08^a^	1.42 ± 0.07^b^^,^ c	1.38 ± 0.11	1.22 ± 0.09^a^	1.26 ± 0.10^a^	1.25 ± 0.07	1.11 ± 0.05^a^	1.09 ± 0.07^a^
CPP (mmHg)	88.9 ± 1.9	74.0 ± 2.1^a^	73.5 ± 2.4^a^	89.8 ± 2.5	71.5 ± 2.8^a^	71.2 ± 3.1^a^	87.5 ± 2.3	70.1 ± 2.2^a^	69.8 ± 2.4^a^
RI	0.53 ± 0.02	0.54 ± 0.02	0.54 ± 0.02	0.51 ± 0.02	0.50 ± 0.01	0.52 ± 0.01	0.52 ± 0.01	0.50 ± 0.02	0.51 ± 0.02
PI	0.82 ± 0.04	0.88 ± 0.06	0.88 ± 0.07	0.76 ± 0.04	0.75 ± 0.03	0.79 ± 0.04	0.77 ± 0.03	0.74 ± 0.04	0.78 ± 0.04

HUT, head‐up tilt; EF, early follicular phase; ML, midluteal phase; MCA, middle cerebral artery; CVR_i_, cerebrovascular resistance index; CPP, cerebral perfusion pressure; RI, resistance index; PI, pulsatility index.

a Significant difference from baseline.

b Main group effect (men vs. ML).

c Main effect of time when compared against ML.

### Hypercapnia

There were no effects of sex, phase, or position on changes in HR in response to hypercapnia (Fig. 4A). Men had an augmented increase in MAP in response to hypercapnia during HUT compared to supine and MAP in men was higher than in EF during HUT (Fig. 4B). All groups had an augmented increase in *Q*_i_ in response to hypercapnia during HUT compared to supine (Fig. 4C). All groups had an augmented increase in SV_i_ in response to hypercapnia during HUT compared to supine (Fig. 4D). Women in both phases had an augmented decrease in TPR_i_ in response to hypercapnia during HUT compared to supine (Fig. 4E). Men also had an augmented decrease in TPR_i_ in response to hypercapnia during HUT compared to supine, but only when compared against ML (Fig. 4E). Men had smaller changes in TPR_i_ due to hypercapnia compared to EF (Fig. 4E).

During HUT, women in both phases had augmented increases in *V*_e_ due to hypercapnia compared to supine and men had an augmented increase in *V*_e_ due to hypercapnia compared to supine when compared against ML (Fig. 5A). All groups had an augmented increase in respiratory rate in response to hypercapnia during HUT compared to supine (Fig. 5B). There were no effects of sex, phase, or posture on changes in *V*_t_ (Fig. 5C). During HUT, all groups experienced an attenuated increase in ETO_2_ in response to hypercapnia compared to supine (Fig. 5D). All groups had an augmented increase in ETCO_2_ in response to hypercapnia during HUT compared to supine (Fig. 5E).

In response to hypercapnia (in both postures), men had smaller increases of diastolic MCA velocity compared to ML (Table 3). Only men had an augmented increase in CPP in response to hypercapnia during HUT (Table 3). Men had higher CPP in response to hypercapnia during HUT compared to EF (Table 3).

**Table 3:** Changes in cerebral hemodynamics in response to hypercapnia in the supine and HUT positions

	Men		Women			
			EF		ML	
	Supine	HUT	Supine	HUT	Supine	HUT
MCA_mean_ (cm/sec)	+12.2 ± 2.0	+12.8 ± 2.1	+13.8 ± 1.38	+16.3 ± 1.5	+16.7 ± 2.8	+16.4 ± 2.3
MCA_systolic_ (cm/sec)	+11.2 ± 2.4	+14.1 ± 2.51	+15.2 ± 1.8	+18.7 ± 1.8	+15.8 ± 2.5	+19.1 ± 2.7
MCA_diastolic_ (cm/sec)	+9.1 ± 2.0^a^	+8.8 ± 2.05^a^	+11.4 ± 1.2	+13.8 ± 1.2	+15.7 ± 3.1	+14.5 ± 1.9
CVR_i_ (mmHg/(cm·sec))	−0.22 ± 0.04	−0.20 ± 0.05	−0.20 ± 0.03	−0.23 ± 0.03	−0.24 ± 0.03	−0.17 ± 0.03
CPP (mmHg)	+0.10 ± 0.58	+4.71 ± 0.87^b^^,^ c	+1.81 ± 0.70	+2.34 ± 0.73	+1.79 ± 0.60	+3.26 ± 0.30
RI	−0.03 ± 0.02	−0.02 ± 0.02	−0.04 ± 0.01	−0.039 ± 0.005	−0.06 ± 0.01	−0.04 ± 0.01
PI	−0.11 ± 0.04	−0.09 ± 0.04	−0.09 ± 0.03	−0.09 ± 0.01	−0.14 ± 0.03	−0.10 ± 0.02

HUT, head‐up tilt; EF, early follicular phase; ML, midluteal phase; MCA, middle cerebral artery; CVR_i_, cerebrovascular resistance index; CPP, cerebral perfusion pressure; RI, resistance index; PI, pulsatility index.

a Main group effect (men vs. ML).

b Significant difference from baseline.

c Group effect between men and EF during HUT.

## Discussion

### Summary

In response to HUT alone, all groups increased HR, *V*_t_, and ETO_2_ while decreasing MAP, *Q*_i_, SV_i_, respiratory rate, ETCO_2_, MCA velocity (mean, systolic and diastolic), CVR_i_, and CPP; only men increased *V*_e_ during HUT. Women in the ML phase had higher *V*_e_ compared to the EF phase at all time points.

In response to CO_2_ (regardless of posture), men exhibited an attenuated decrease in TPR_i_ compared to the EF phase, that is, men experienced less peripheral vasodilation during hypercapnia, and men exhibited smaller increases of diastolic MCA velocity compared to the ML phase, that is, men experience less cerebrovascular dilation during hypercapnia.

In response to hypercapnia in the HUT position, all groups experienced augmented increases of *Q*_i_, SV_i_, *V*_e_, respiratory rate, and ETCO_2_ while experiencing augmented decreases of TPR_i_ and ETO_2_; only men had an augmented increase of MAP (and thus CPP). There were no effects of menstrual cycle on the CO_2_ chemoreflex response in HUT.

### Head‐up tilt

We hypothesized that: (1) during upright tilt, women would have lower MAP, lower brain blood velocity, and lower respiratory rate compared to men; and (2) women in the ML phase would have greater cerebrovascular reactivity to CO_2_ compared to EF. Our results did not support these hypotheses.

Despite the fact that young women are known to be more susceptible to orthostatic intolerance, all groups experienced a decrease in MAP during HUT. Interestingly, Fu et al. (2009) observed that only women experienced gradual decreases in systolic blood pressure during 60° HUT; however, these trials lasted for 40 min and a decrease in pressure was not observed until >5 min of tilt. Similarly, in the current study all groups experienced equivalent reductions in MCA velocity and CVR_i_ during tilt implying cerebrovascular vasodilation in all groups during HUT; however, during a longer investigation of orthostatic stress (i.e., 10 min of standing), Abidi et al. (2017) found that men increased CVR_i_ indicating cerebral vasoconstriction. Therefore, future studies should use longer periods of orthostatic stress in order to determine sex/menstrual cycle differences in the hemodynamic and cerebrovascular responses to orthostatic stress.

Women during the ML phase had higher *V*_e_ than women in the EF phase at all time points. Dombovy et al. (1987) also found higher *V*_e_ in women during the luteal phase compared to the follicular phase and attributed it to higher progesterone levels in the luteal phase. Indeed, the increase in *V*_e_ due to progesterone is possibly mediated by an upregulation of progesterone receptors in the hypothalamus (Bayliss et al. 1991). Furthermore, female cats injected with progesterone display increased phrenic nerve activity compared to males allowing for hyperventilation (Bayliss et al. 1987). The enhancement of *V*_e_ by progesterone could also help to explain why no baseline ventilatory difference was seen between men and women during the ML phase.

All groups experienced an increase of *V*_t_ and a reduction of respiratory rate with HUT. The reduction of respiratory rate in men was unexpected since Abidi et al. (2017) found that only women decreased respiratory rate during 10 min of standing. However, in the current study, we found that activating our flow‐through gas system for delivery of normoxia decreased respiratory rate in men. Therefore, this reduction of respiratory rate in men may be an artifact of breathing from a compressed air tank rather than room air. We suggest that this reduction of respiratory rate could be a result of inappropriately matched airflow in men leading to reduced respiratory rate. Women also experienced higher ET‐O_2_ and lower ET‐CO_2_ during normoxia administration potentially indicating that the supplied air flow was too high, that is, the delivered airflow diluted the expired gases. We recommend that individual inspiratory flow rates are measured prior to testing when using a flow‐through gas system. Despite all groups increasing *V*_t_ and decreasing respiratory rate during upright tilt, only men had an increase of ventilation. These results confirm those of previous studies who found increased ventilation during orthostatic stress in men and mixed sex groups (Gisolf et al. 2004; Wang et al. 2004; Chang et al. 2005). An increase of ventilation in men would normally be expected to decrease end‐tidal CO_2_ and increase end‐tidal O_2_ more than women due to greater gas exchange (as previously observed by Serrador et al. 2006); however, this was not observed. We suggest that a reliance on abdominal breathing in men (Romei et al. 2010), and therefore movement of splanchnic blood pools (Aliverti et al. 2009), together with an enhanced respiratory pump leads to greater return of hypercapnic and hypoxic blood from the periphery. We further suggest that future studies should investigate sex differences in the Hering–Breuer reflex. Since men have larger lungs, the pulmonary stretch afferents could be less sensitive allowing for greater inflation of the lungs compared to women.

### Hypercapnia

We hypothesized that: (1) CO_2_ chemoreflex function would be enhanced in the upright posture compared to supine in both sexes, and (2) women during the EF phase would have enhanced CO_2_ chemoreflex activity in response to hypercapnia during HUT compared to the ML phase and men. The first hypothesis was supported by our findings but not the second. The results of this study partially confirm those of Kastrup et al. (1997) who found greater cerebrovascular reactivity to CO_2_ in women compared to men. In the current study, only women in the ML phase of the menstrual cycle had a greater increase of diastolic MCA velocity during hypercapnia compared to men. This could be due to the higher concentration of either estrogen or progesterone in the ML phase (which is low in the EF phase) since both hormones can act as vasodilators (Kawano et al. 1997; Patkar et al. 2011; Ramírez‐Rosas et al. 2014). However, contrary to our hypothesis, cerebrovascular reactivity to CO_2_ was not different between the EF and ML phases of the menstrual cycle, suggesting that the higher concentrations of estrogen and progesterone in ML do not influence cerebrovascular reactivity to CO_2_. Furthermore, Peltonen et al. (2016) investigated the cerebrovascular response to hypercapnia between the EF and late follicular phases of the menstrual cycle, comparing low to high estrogen, respectively, and found no difference between phases. Future studies are needed to compare the late follicular and ML phases in order to investigate the role of higher concentrations of progesterone.

All groups had significantly augmented increases in *V*_e_ due to hypercapnia during HUT indicating greater CO_2_ chemoreflex function. This augmented function could be due to interactions with other autonomic reflexes such as the baroreflex. Taneja et al. (2011) found that baroreflex unloading during upright posture did not affect the sensitivity of the CO_2_ chemoreflex in a healthy control population; however, they investigated the effect of hypercapnia with concurrent hyperoxia, thus perhaps augmenting the CO_2_ chemoreflex while inactivating the peripheral O_2_ chemoreflex. This increase of *V*_e_ in all groups was driven by a higher respiratory rate in hypercapnic tilt which could have also contributed to the increases of SV_i_ and *Q*_i_ via greater respiratory pump action. Greater respiratory pump action could also increase venous return from the periphery and thus increase ETCO_2_ and decrease ETO_2_, as observed. Alternatively, higher sympathetic outflow during hypercapnic tilt could play a role. While we do not know if there were sex differences in the sympathetic response to hypercapnia in HUT, higher sympathetic activity would be expected to augment the blood pressure response and attenuate vasodilation in men (which is what we observed). However, Hart et al. (2009, 2011) observed that no relationship exists between muscle sympathetic nerve activity and TPR in young women, and suggests that in women increased β2‐adrenergic vasodilatory responses may offset α‐adrenergic vasoconstriction during a sympathetic stimulus (i.e., HUT). Therefore, the greater blood pressure response to hypercapnia in HUT in men could be due to either greater sympathetic output or greater neurovascular transduction of the sympathetic output.

### Limitations

All female participants in this study self‐reported menstrual cycle phase defining day 0 as the first day of menstruation; however, we did not confirm plasma concentrations of estrogen or progesterone in the ML phase of the menstrual cycle. Future studies should include this or urinary testing of luteinizing hormone to confirm ovulation.

Direct measurements of peripheral blood flow and muscle sympathetic nerve activity were not included as part of this study. These additions would have allowed for firmer conclusions pertaining to changes in sympathetic output and/or neurovascular transduction. Similarly, measurements such as central venous pressure or inferior vena cava diameter could have been implemented as indices of venous return. Furthermore, MCA velocity was used as an index of brain blood flow. However, hypercapnia has been shown to vasodilate large intracranial arteries (Coverdale et al. 2014; Verbree et al. 2014; Mikhail Kellawan et al. 2016), indicating that the increase of brain blood flow observed during hypercapnia could have been greater than that indicated by velocity alone. Additionally, Lewis et al. (2015) observed that during orthostatic stress the decrease of MCA velocity is smaller than the decrease of combined carotid and vertebral flow. Therefore, decreases in MCA velocity during upright posture may underestimate decreases in brain blood flow. Sex differences in these effects are unknown.

Measurements of brain blood flow velocity were taken in the second minute of gas administration in order to minimize the total duration of upright tilt per person due to multiple trials. However, a recent study by Hoiland et al. (2017) found that the greatest increase of brain blood flow velocity in response to steady‐state CO_2_ (+9 mmHg) takes ~3 min to occur. Therefore, the maximal vasodilatory effect of the hypercapnic gas administration could have been missed.

## Conclusions

We have provided evidence that there are no sex or menstrual cycle differences in the cerebrovascular responses to tilt; however, only men exhibit an increase of ventilation during tilt perhaps contributing to a greater respiratory pump effect and thus a mechanism for greater venous return and protection of MAP. Furthermore, we have shown that the CO_2_ chemoreflex is augmented in both men and women during HUT.

## Conflict of Interest

Authors have no conflicts of interest or competing interests.
